# Attitudes and experiences of registered diabetes specialists in using health apps for managing type 2 diabetes: results from a mixed-methods study in Germany 2021/2022

**DOI:** 10.1186/s13690-023-01051-0

**Published:** 2023-03-07

**Authors:** Julian Wangler, Michael Jansky

**Affiliations:** Center for General Medicine and Geriatrics, University Medical Center of the Johannes Gutenberg University Mainz, Am Pulverturm 13, 55131 Mainz, Germany

**Keywords:** Health apps, mHealth, Prevention, Health promotion, Diabetes care

## Abstract

**Background:**

Hardly any area of application for health apps is seen to be as promising as health and lifestyle support in type 2 diabetes mellitus. Research has emphasised the benefits of such mHealth apps for disease prevention, monitoring, and management, but there is still a lack of empirical data on the role that health apps play in actual type 2 diabetes care. The aim of the present study was to gain an overview of the attitudes and experiences of physicians specialising in diabetes with regard to the benefits of health apps for type 2 diabetes prevention and management.

**Methods:**

An online survey was conducted amongst all 1746 physicians at practices specialised in diabetes in Germany between September 2021 and April 2022. A total of 538 (31%) of the physicians contacted participated in the survey. In addition, qualitative interviews were conducted with 16 randomly selected resident diabetes specialists. None of the interviewees took part in the quantitative survey.

**Results:**

Resident diabetes specialists saw a clear benefit in type 2 diabetes-related health apps, primarily citing improvements in empowerment (73%), motivation (75%), and compliance (71%). Respondents rated self-monitoring for risk factors (88%), lifestyle-supporting (86%), and everyday routine features (82%) as especially beneficial. Physicians mainly in urban practice environments were open to apps and their use in patient care despite their potential benefit. Respondents expressed reservations and doubts on app user-friendliness in some patient groups (66%), privacy in existing apps (57%), and the legal conditions of using apps in patient care (80%). Of those surveyed, 39% felt capable of advising patients on diabetes-related apps. Most of the physicians that had already used apps in patient care saw positive effects in increased compliance (74%), earlier detection of or reduction in complications (60%), weight reduction (48%), and decreased HbA1c levels (37%).

**Conclusions:**

Resident diabetes specialists saw a real-life benefit with added value from health apps for managing type 2 diabetes. Despite the favourable role that health apps may play in disease prevention and management, many physicians expressed reservations regarding usability, transparency, security, and privacy in such apps. These concerns should be addressed more intensively towards bringing about ideal conditions for integrating health apps successfully in diabetes care. This includes uniform standards governing quality, privacy, and legal conditions as binding as possible with regard to apps and their use in a clinical setting.

**Supplementary Information:**

The online version contains supplementary material available at 10.1186/s13690-023-01051-0.

## Background

More than 500 million people contracted diabetes across the world in 2020, including around 8 million in Germany [1–3]. Of these, 95% were suffering from type 2 diabetes mellitus with widespread weight gain and lack of exercise as major reasons [1–3]. Patients with type 2 diabetes also often have other cardiovascular risk factors in addition to chronically high blood sugar and should always agree on individual nutrition, bodyweight, blood sugar, blood pressure and lipid status targets with their physicians [4–9]. As clinical guidelines recommend, the main focus should be placed on adopting a healthy lifestyle.

Health apps for the purpose of assisting in monitoring and management are seen to be especially promising in type 2 diabetes mellitus as a lifestyle-induced disease [10–12]. According to the World Health Organization (WHO), mobile health (mHealth) apps are defined as software programs that, in the sense of personal digital assistants, run on smartphones or tablet platforms, aiming at enabling and improving the delivery of health care. Such tools can promote health and primary disease prevention or support people with chronic illnesses in managing their medical conditions or improve treatment adherence [10, 11]. Depending on the functionality and focus, apps specifically intended for diabetes may fulfil a variety of roles including management data documentation such as vital parameters, logging measured values, diet and nutrition information, calorie and exercise profile creation, and regular blood sugar measurement and medication intake reminders and tracking [13–15].

Various studies have shown diabetes apps to have beneficial effects on disease management [10, 11], especially towards increasing exercise levels, healthy nutrition, weight reduction, and stress management [16–22]. Favourable outcomes with respect to compliance and psychological well-being have also been demonstrated [23, 24]. Kebede et al. reported improvement in self-management, information, and motivation in type 2 diabetes patients using these apps [25]. One meta-analysis reported an improvement in metabolism in type 2 diabetics after app usage [26]. Veaezie examined 15 studies on apps used in diabetes management [27], reporting a significant decrease in HbA1c levels. One meta-analysis on 13 randomised controlled studies showed a significant reduction in HbA1c levels in six of the studies [28]. Offringa et al. reported that using mobile apps led to improved glucose control with less frequent hyperglycaemic episodes [29].

Attitudes amongst physicians specialising in diabetes towards health apps for managing type 2 diabetes plays a decisive role even considering the initial empirical evidence [17]. Depending on attitudes, prior knowledge and experience, physicians may recommend specific apps to their patients and regularly use these apps in their healthcare strategy. Several analyses have implied that medical recommendations may increase usage levels and compliance in using apps focused on diabetes management by between 10 to 30 % [30].

## Methods

### Study aims and design

The present exploratory study focuses on health professionals, and not patients. It served the purpose of gaining an overview of the attitudes and experiences of physicians in specialist diabetes practices with regard to the benefits of health apps for type 2 diabetes prevention and management. This involved identifying potential added value and issues in clinical settings as well as observations in healthcare, including recommendations for specific apps and their effectiveness. Conclusions from these findings should assist in applying these potential benefits from health apps in type 2 diabetes mellitus diagnosis, management, and prevention.

The present multi-part study combined quantitative and qualitative components for adequate coverage of the topic. First, a comprehensive online survey amongst all physicians in practices specialised in diabetes in the Federal Republic of Germany was performed. After the above survey, a smaller qualitative study was performed in order to obtain additional knowledge by means of interviews with resident diabetes specialists.

### Study participants and ethics approval

During this study, no sensitive patient data was gathered or clinical tests performed. This is a strictly anonymized survey of a total of 538 doctors from specialist diabetology practices. The Ethics Commission of the State of Rhineland-Palatinate, Germany, informed us that approval by an ethics committee was not necessary.

A total of 1746 general practitioners and internists were identified in 1041 practices using the online physician locator provided by the Association of Statutory Health Insurance Physicians in each federal state. This roughly corresponded to the number reported in publicly available healthcare statistics in Germany [31, 32].

All the physicians identified were sent postal invitations to take part in the anonymous survey between September 2021 and April 2022. A once-only letter explained the intention to conduct a survey as well as its anonymisation, giving the physicians to be surveyed password-protected access to the online survey amongst other things. Participants were not given any remuneration or incentives.

The survey saw 543 questionnaires returned from the 1746 sent. The 538 fully completed questionnaires were included in analysis. This yields a response rate of 31%. The sample population was as follows:

- Gender: 50% male, 50% female
- Mean age: 55, Median: 55
- Practice setting: 52% in medium-sized and large towns or cities, 48% in small towns or rural areas
- Practice model: 36% individual practices, 59% joint practices, 5% other
- Patients per quarter: Up to 1000: 14%, 1001 to 1500: 28%, 1501 to 2000: 25%, more than 2000: 33%

Sixteen semi-standardised interviews were also performed with randomly selected resident diabetes specialists distributed throughout Germany between April and June 2022. Nine of these interviews were taken by telephone, seven in person. None of the interviewed diabetes specialists took part in the quantitative survey.

### Data collection and questionnaire

The survey questionnaire (see Additional file 1) used was drawn up using extensive literature research on the one hand, and three preliminary studies targeting general practitioners on potential areas of use for health apps on the other [17, 20, 33]. This involved adopting questions on general acceptance, experience, and willingness to use the apps, and usage potential in actual situations in relation to diagnostics, treatment and prevention of chronic diseases then specifically tailored to type 2 diabetes mellitus.

The exploratory questionnaire consists of a total of 23 questions with the above-mentioned focuses. The results of the preliminary studies were primarily incorporated into the creation of the item batteries used (questions 13, 14, 16, 17, 21).

In the questionnaire, mostly closed questions were asked, which could be answered either by single choice (questions 1–4, 8–12, 15, 18–20, 22) or multiple choice (questions 7, 13, 14, 16, 17, 21, 23). Some of these questions included an optional text field for additional answers (questions 7, 16, 17, 21, 23). Besides, two open-ended questions were asked (questions 5, 6).

In order to achieve a good compromise between data quality and intuitive answerability of the questionnaire for the time-critical target group of diabetologists, ordinal scales were widely used (usually with four levels, questions 2–4, 9–12, 15, 18–20).

Sociodemographic data collected included gender, age, practice location population, establishment model and number of patients per quarter. The validity and reliability of the questionnaire was evaluated in the course of a pretest carried out on 20 resident diabetes specialists. Cronbach’s alpha was calculated to assess the reliability and it was 0.82.

The qualitative interview survey instrument (see Additional file 2) was drawn up using the online survey as a basis with the aim of developing on the quantitative findings to greater depth. In essence, the development of the interview guideline was about converting some of the closed questions of the questionnaire into open questions in order to be able to explore the topic additionally. The main focus areas of the interview guideline were: clinical picture of type 2 diabetes and its significance in everyday practice; perception and significance of health apps in general; health apps with regard to type 2 diabetes mellitus; own experiences using type 2 diabetes health apps. Three interviews were conducted in advance with physicians from practices specialising in diabetes towards further specifying and validating the interview guideline used.

### Data analysis

SPSS 23.0 was used for data analysis. Student’s *t*-test for independent samples was used to detect significant differences between two groups. Values of *p* < .001 were considered highly significant.

The team evaluated the resulting transcripts from the qualitative interviews using qualitative content analysis according to Mayring [34] (MAXQDA Software) after data collection. Our focus lay on forming logical categories from the various opinions and experiences. Selected citations are presented to support the quantitative findings.

We used STROBE for reporting statement purposes.

## Results

### Usage potential of health apps for managing type 2 diabetes

51% of the respondents gave health apps a favourable rating as potential aids in healthcare. A quarter of the sample were sceptical (24%) or undecided (25%) on these mHealth applications. Physicians in cities and medium-sized towns viewed apps more favourably than those in small towns and rural communities (60% vs. 34% favourable ratings, *p* < .001). Respondents below the average age of 55 were also more open to apps than older physicians (55% vs. 41% favourable ratings, *p* < .001).

Most physicians anticipated the benefit health apps could mean for prevention, diagnostics, and management in type 2 diabetes mellitus patients as very important (12%) or rather important (44%) as opposed to 42% not so important and 2% no benefit. Most respondents from urban environments rated the benefit of apps significantly more favourably than those in a small-town or rural environments (64% very/rather important vs. 42% very/rather important, *p* < .001).

*“I think we should be looking at the use of apps in a broader disease management context. Apps could clearly offer added value if they can be integrated into regular medical care, in a fixed disease management programme for example.”* (I-2 m).

88% the respondents thought health apps would be very or rather useful in prevention (such as in for self-monitoring of risk factors). 86% thought health apps wold be useful in maintaining a health-promoting lifestyle (such as diet and exercise). 82% said they would appreciate apps helping patients in type 2 diabetes patients management (such as by giving reminders to take medication or vaccinations, or to go to check-ups). 65% saw monitoring and treatment in chronic disease as a useful area of application.

*“No doubt, these eHealth tools are definitely useful for long-term modifications in lifestyle and everyday habits. There is a lot to be said for them in my experience. Even so, selecting the right apps is important, and queries from patients should be expected.”* (I-11f).

**Fig. 1:**
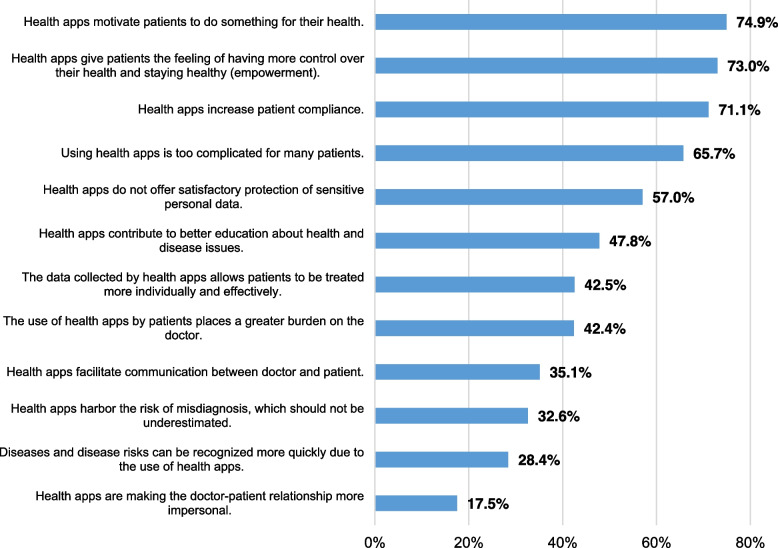
Responses to statements regarding health apps and their use in treating patients with type 2 diabetes mellitus (*N* = 538; online survey amongst all physicians at practices specialised in diabetes in Germany, survey period: 2021/2022)

Many of the respondents considered reinforcing patient motivation as a potential benefit in these apps (see Fig. 1). Some members in the sample also associated app usage with improvement in patient education and more effective treatment. However, a large share of the sample saw apps as being too complicated for many patient groups. They also expressed concern that incorrect health data could be collected or, in extreme cases, that treatment strategies would fail. Most were dissatisfied with data privacy in most of the health apps, and many respondents were concerned about the additional workload involved.

### Personal experience with regard to the use type 2 diabetes apps

16% of the respondents stated that they had seen many patients send their health data such as blood sugar logs collected from health apps to the practice in digital form (17% some, 41% rather few, 26% none). Respondents below the average age saw significantly more of these patients than older physicians (many/some: 48% vs. many/some: 8%, *p* < .001).

Half the respondents stated that their own patients frequently (10%) or occasionally (42%) asked them about health apps for type 2 diabetes mellitus prevention and/or management (38% rarely, 10% never). Most diabetes specialists reported raising the subject of these apps to their patients often (10%) or occasionally (36%). Respondents stating that they were more often asked about apps or that they raised the subject themselves were mostly physicians in urban areas.

*“From my experience, you can’t expect patients to deal with these apps on their own. A coherent healthcare plan plays a crucial role. Accordingly, it makes sense for doctors to make the first move and raise the topic of specific apps with their regular patients. This is the only way of ensuring that the app will be used.”* (I-5 m).

The same applies to recommendations for specific apps. In this respect, 48% stated recommendations for a specific app were frequent (14%) or occasional (34%) compared to rare (22%) or never (30%). The lion’s share of these respondents (47%) stated that they had already used and recommended the mySugr app in their patient care. Other apps named included FreeStyle Libre (28%), Dexcom G6 (15%), Accu-Chek Connect (9%), and DiabetesPlus (8%).

In an open question the respondents were asked to name important criteria as prerequisites for recommending an app. As the results show, the criteria mentioned mainly included simplicity, accessibility, comprehensibility, and intuitive use, but also data privacy and security, exercise motivation, and a sound foundation in guidelines and evidential medicine.

*“Evidence-based criteria in an app are an important quality indicator for me. This means that the app has been tested and has, for example, been mentioned in recommendations from professional associations or other care-related organisations”* (I-16 m).

*“There is probably still a general lack of broad review based on uniform and transparent criteria for apps available in Germany that would warrant recommendation without reservation. [...] The potential for apps to inspire patients towards a change in lifestyle should not be underestimated. So gamification is an important factor in everyday use.”* (I-3f).

A clear preference for certain sources of information emerged amongst physicians recommending apps. Of these, 73% stated that they obtained their information on mHealth apps such as evaluation and recommendation of new apps from the German Diabetes Association (DDG) website. Other sites such as HealthOn (14%), Telemedicine Competence Centres of the federal state (17%), Federal Institute for Drugs and Medical Devices (13%), and the National Health Portal (7%) were used much less frequently for information.

As far as the respondents were aware, their type 2 diabetes patients mainly used health apps for prevention and self-monitoring (82%) and to keep to a healthy lifestyle (78%). Well over half of the respondents at 58% cited monitoring and management (such as documenting parameter trends and symptoms) and 51% for medication and blood sugar measurement reminders.

### Perceived benefit of type 2 diabetes health apps

34% of those surveyed assume that health apps can play a very important (10%) or rather important (24%) role towards faster disease detection and diagnosis, while 46% saw limited or minor benefit (15% no benefit, 5% could not say).

One question was aimed at identifying those clinical conditions that could be more effectively identified by health apps in the experience of the respondents. 65% said they expected or observed hypoglycaemia to be detected more quickly by using a health app. 38% named episodes of depression, 35% metabolic syndrome, 19% hyperosmolar coma, and 17% named diabetic foot syndrome and diabetic neuropathy each.

**Fig. 2:**
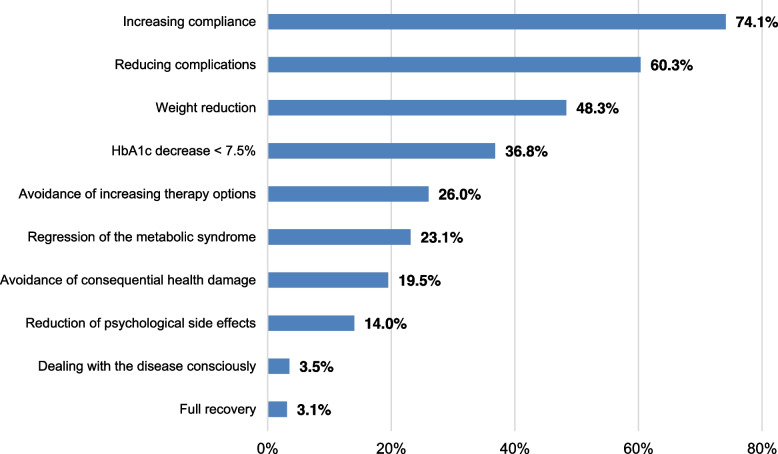
Which of the following effects have you already seen from your type 2 diabetes mellitus patients successfully using health apps? (*N* = 538; online survey amongst all physicians at practices specialised in diabetes in Germany, survey period: 2021/2022)

Most respondents observed an increase in compliance and a reduction in complications such as hypoglycaemia as favourable results of successful app use (see Fig. 2). Around half stated that their patients had lost weight as a result of using the app. Every third respondent had already observed a decrease in HbA1c levels to below 7.5%. Less frequent improvements included a reduction in the metabolic syndrome, prevention of sequelae and reduction in psychological side effects.

### Issues and potential for optimisation

Respondents gave a rather reserved rating on their awareness and knowhow regarding the general range of health apps available for type 2 diabetes mellitus prevention and/or management. Under a third at 30% rated their awareness of the range of apps available as (very) extensive compared to 70% as rather or very restricted.

43% of the respondents saw themselves as capable of distinguishing good from bad health apps for type 2 diabetes mellitus prevention and/or management; significantly more of these physicians were in urban rather than rural practice settings (55% vs. 27%, *p* < .001). 39% of the respondents saw themselves as capable of giving patients thorough and competent advice on specific apps in this field of specialisation, again with significantly more of them in urban rather than rural practice settings (47% vs. 21%, *p* < .001).

*“This is a highly dynamic and chaotic issue. There are new apps constantly appearing as well as updates and other changes. Physicians can’t be expected to keep up on all the new developments all the time, not even in an isolated specialisation area like type 2 diabetes mellitus. In other words, we need more consistent support from other players. Solid information sources from independent testers, and obviously more involvement from specialist bodies.”* (I-7 m).

**Fig. 3:**
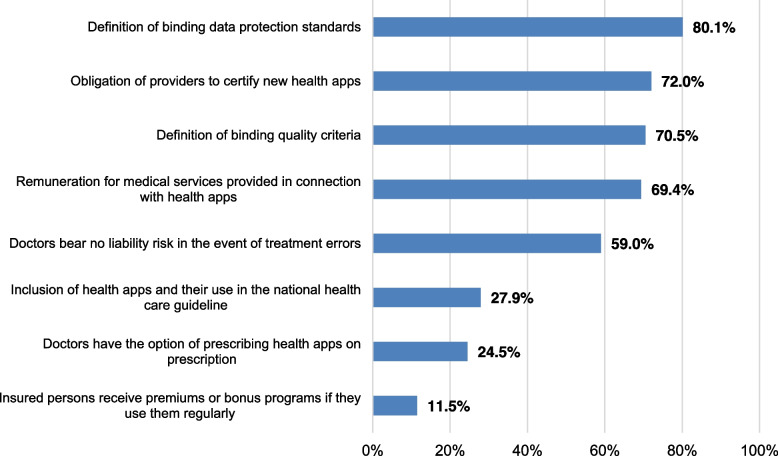
Suggestions for optimising health apps intended for use in diabetes mellitus type 2 (*N* = 538; online survey amongst all physicians at practices specialised in diabetes in Germany, survey period: 2021/2022)

A clear majority of the respondents expressed a desire for authoritative data privacy and quality standards to be defined and enforced towards more attractive health apps for use in type 2 diabetes mellitus (see Fig. 3). Most proposed mandatory certification in new apps. The matter of payment for medical services provided in connection with health apps was also raised, such as a separate position in the fee schedule. Apart from that, physicians expressed a need for legal issues to be clarified in including apps in healthcare.

*“What makes me personally reluctant is that I can’t ever be a hundred percent sure how much I can rely on an app for the patients I treat. This begins with data collection and continues with everyday app use by patients. Could I be held liable for any bugs found in the app? Or for patients using the app incorrectly because of a misunderstanding on how to use it? At what point should I start worrying? How much involvement in app support is legitimate, and where do I start taking dangerous risks? […] I don’t see enough regulation or public communication on this.”* (I-8w).

Most respondents said they would in principle be willing to include health apps in their patients’ healthcare far more (26%) or rather more (54%) than today if National Healthcare Guideline (NVL) specifically addressed the use of health apps with regard to type 2 diabetes mellitus prevention, monitoring, and management.

## Discussion

### Main findings and comparison with prior work

The results demonstrate that most physicians in practices specialising in diabetes do see potential benefits of health apps for managing type 2 diabetes. Such benefits include effective reinforcement of empowerment, motivation and compliance, and also reminder and lifestyle-supporting features in disease management towards improved type 2 diabetes patients prevention and management [35, 36]. Earlier studies have demonstrated recognition amongst general practitioners and specialists as to specific usage scenarios and added value in apps [17, 20]. Younger physicians, especially in urban environments, show a favourable attitudes towards health apps and use these tools in everyday practice – a result that agrees with other studies on the subject [12, 18–20].

Even so, a substantial share of the respondents expressed scepticism about health apps that included user-friendliness and reliability in existing apps, legal issues such as liability, and the resulting additional workload from additional responsibility in patient counselling. A substantial number of respondents were also concerned about incorrect app use or vital parameter measurement causing risks such as misdiagnosis. The distinct scepticism regarding data privacy in health apps is remarkable considering the strict data protection standards by international comparison due to the requirements set in the European General Data Protection Regulation (GDPR), which applies in Germany. Even so, respondents showed a considerable level of insecurity on this point in particular.

Most have admitted a lack of sufficient general awareness on topic-related health apps owing to the huge and dynamic range of apps available, lack of transparency and basic orientation; this results in limited confidence in advising patients [12, 13, 17, 20, 37].

Respondents that recommended apps named various criteria that would need to be met before making specific recommendations. These criteria mainly included ease of use or usability, reliability, and data privacy guarantees.

Physicians already using apps in patient care had observed positive effects in increased compliance, earlier detection of or reduction in complications, weight reduction, and decreased HbA1c levels. These findings tally with those of other studies [38–41]. Overall, a number of studies have shown digital applications can significantly contribute to a substantial reduction in HbA1c levels, consistent blood sugar management, improved wellbeing, and lifestyle compliance [16–22, 25, 42–49]. Self-monitoring has also been found to result in a decrease in cardiovascular mortality with increased awareness of symptoms [42].

Besides, studies have reported forgetting to take medications and changing doses as reasons for poor compliance in chronic diseases. One such study examined seniors and their medication-taking behaviour using the MyTherapy app for self-monitoring their type 2 diabetes [24]. These seniors used the app most frequently for reminders to measure blood glucose levels and take medication, followed by exercise reminders. Using the app resulted in an improvement in psychological well-being and medication compliance. Other studies have shown the MyTherapy app to increase compliance and strengthen doctor-patient relationships in type 2 diabetes patients [23, 50].

Patient surveys have demonstrated people living in rural areas to be less accepting of health apps in general. However, these populations could benefit even more from telemedical data transmission than urban populations due to the greater distances to physicians and increasing decline in outpatient care providers in some regions [10, 16, 18, 42]. Some respondents were also aware of this by recognising possibilities for easier, low-threshold communication, strengthening the doctor-patient relationship and other benefits.

Most respondents expressed a wish for authoritative data privacy and quality standards as well as certification for new apps, requirements scientifically established in other studies including the CHARISMA study in particular [10, 11]. Many respondents saw the benefit of the national healthcare guideline explicitly addressing evidence-based use of health apps for type 2 diabetes prevention, monitoring, and management. Most respondents stated that they could imagine including health apps in patient care more intensively than before if all these conditions had been met. Other study results have also shown there to be a great desire for evidence-based integration of health apps [50–52].

Physicians in Germany have been permitted to prescribe patients approved digital health applications (so called DiGAs) since the summer of 2020 - a step that was unique in the world at that time and still is [53]. Since then, physicians have been able to prescribe DiGAs to patients with costs covered by the national health system. DiGAs are set to make disease diagnostics and recognition more effective, provide support in treatment, and contribute to prevention while guaranteeing high quality standards. In contrast to ordinary, freely available health apps, these prescription apps are certified as medical devices. DiGAs are reviewed in detail using a standard procedure defined by the German Federal Institute for Drugs and Medical Devices (BfArM). This requires manufacturers to apply for approval in the course of an audit process on a variety of requirements (CE markings for medical devices, data protection, security standards, information quality, usability and robustness in operation, patient safety). They are also required to provide sufficient documentation for the added value of the application in its effect on healthcare.

The range of such approved health apps is still rather limited, but diabetes-related apps in particular have become more strongly represented (apps such as Zanadio, Oviva Direkt, ESYSTA, VIDEAmellitus) [54]. These prescription health apps have been tested for effectiveness based on quality standards with approval for clearly defined specialisations, so they stand to boost willingness amongst not only diabetes patients but also their doctors in the long term. Medical concerns as reflected in the present survey may therefore be addressed far more effectively by prescription apps [55]. Initial surveys of doctors on approved digital health applications show that they are rated as significantly more trustworthy and reliable than conventional health apps. This offers new opportunities and application potential for the future integration of health apps into patient care [56].

### Strengths and limitations

We recruited a heterogeneous sample of physicians from practices specialising in diabetes. Even so, the present study cannot lay any claims to being representative due to the limited number of respondents.

Moreover, it cannot be ruled out that physicians with favourable attitudes and experiences towards health apps took part in the survey to a greater extent than those with negative experiences (possible selection bias).

The present exploratory survey primarily served towards gauging general opinion rather than performing a more nuanced analysis of clinical effectiveness amongst mHealth apps. The breadth and complexity of health apps as a topic limited the present study to an initial exploratory approach. The authors see a need for further, more empirically grounded intervention studies focusing on specific application and use opportunities, but also weaknesses of health apps in healthcare for type 2 diabetes patients.

## Conclusions

The results indicate that diabetes specialists perceive the added value of type 2 diabetes health apps in prevention, diagnostics, and management, and have already had favourable experiences in the selective use of apps in healthcare. Even so, many physicians still harbour concerns about transparency, security and privacy, and user-friendliness in these apps [10, 11, 17, 20]. In addition, general awareness and selection of possible apps pose issues. This has limited willingness to recommend or consistently use these digital applications in everyday practice.

The CHARISMA study brought together a variety of measures towards optimising health apps [11]. These measures included app manufacturers being required to observe authoritative quality criteria, general quality control, and the establishment of clear criteria for intended use in each app. Whether doctors can be held liable for treatment errors caused by app data remains unclear. The German government has made it possible for physicians to prescribe health apps as medical devices, which could make it more of a challenge for manufacturers to achieve quality standards in the future; this could lead to a favourable transformation of the app market in the longer term [53].

General awareness and orientation are a current issue; this means that evidence-based guidance from diabetes networks and relevant specialist associations could help physicians gain a qualified overview of the plethora of apps in this specialisation, keep up to speed on current developments, and assess which app would be best suited to which specialism [16]. The authors also see a need for more training courses explaining the possibilities and limitations of using apps in medical practices and introduce the integration of apps into patient counselling.

A clear framework and larger evidence base will prove crucial in minimising the concerns of diabetes specialists and helping to educate physicians with studies on the benefits, opportunities, and risks of health apps. User acceptance from both patients and physicians will only increase once physicians gain confidence in their application. Under these conditions, high-quality health apps could become a staple in healthcare as they develop their potential in type 2 diabetes patient care.

## Supplementary Information

**Additional file 1.** Questionnaire.

**Additional file 2.** Interview guideline.

## Data Availability

All data generated or analysed during this study are included in this published article.
