# Supplementary material for: Attitudes and experiences of registered diabetes specialists in using health apps for managing type 2 diabetes: results from a mixed-methods study in Germany 2021/2022
Source: Arch Public Health. 2023 Mar 7;81:36. doi: 10.1186/s13690-023-01051-0 (PMC9990333; doi:10.1186/s13690-023-01051-0)
Supplement: Supplementary file 1 — Additional file 1. Questionnaire. [file 13690_2023_1051_MOESM1_ESM.docx]

## **Additional file 1: Questionnaire**

1. **Do you think that health apps are generally a good thing, or are you more sceptical about them?**

⃝ Approve ⃝ Sceptical ⃝ Undecided

1. **How often do your patients ask you about health apps for type 2 diabetes mellitus prevention and/or management?**

⃝ Often ⃝ Occasionally ⃝ Rarely ⃝ Never ⃝ Can’t say, don’t know

1. **How often do you mention apps for type 2 diabetes mellitus prevention and/or management to your patients?**

⃝ Often ⃝ Occasionally ⃝ Rarely ⃝ Never ⃝ Can’t say, don’t know

1. **How often do you recommend specific apps for type 2 diabetes mellitus prevention and/or treatment to patients?**

⃝ Often ⃝ Occasionally ⃝ Rarely ⃝ Never ⃝ Can’t say, don’t know

1. **Which apps, specifically?**

1. **Please name three to five criteria you find especially important in recommending a specific health app to patients. What particular features do you think a health app needs to have?**

1. **Which of the following platforms do you use for information on health apps?**

⃝ Health on

⃝ German Diabetes Association (DDG)

⃝ German Health Information Systems Action Forum (afgis)

⃝ State Telemedicine Competence Centre (ZTG)

⃝ German Federal Institute for Drugs and Medical Devices (BfArM)

⃝ App-synopsis (University of Braunschweig-Hanover Institute for Medical Informatics)

⃝ US Food and Drug Administration (FDA)

⃝ German National Health Portal (gesund.bund.de)

⃝ Other, please state:

1. **Please give an estimate: How many patients with type 2 diabetes mellitus have you treated at your practice in the past twelve months?**

⃝ Around _______ patients ⃝ Don’t know ⃝ No answer

1. **Regarding your patient base as a whole: How would you rate your patients’ willingness to use health apps in type 2 diabetes mellitus prevention and management?**

⃝ Very high ⃝ Rather high ⃝ Rather low ⃝ Little or no willingness at all

1. **Do you have patients with type 2 diabetes mellitus sending data such as blood sugar logs collected by a health app to your medical practice in digital form, such as e-mail?**

⃝ Yes, many ⃝ Yes, some ⃝ Yes, a few ⃝ No, none

1. **How far do you think health apps can assist patients with type 2 diabetes mellitus in disease prevention, diagnostics, and management? What kind of benefit do you think health apps bring to the table?**

⃝ Very favourable ⃝ Rather favourable ⃝ Not very favourable ⃝ No benefit

1. **In what areas do you think health apps are useful, and where could they benefit patients with type 2 diabetes mellitus? (You may choose multiple options)**

**Prevention, such as in patient self-monitoring for risk factors – bodyweight, blood pressure, blood sugar and similar – and health data such as steps taken, fluid intake volumes, diet and so on.**

⃝ Very useful ⃝ Somewhat useful ⃝ Not very useful or not useful at all ⃝ Difficult to say

**Monitoring and treating chronic diseases such as diabetes, hypertension, CHD, and their sequelae**

⃝ Very useful ⃝ Somewhat useful ⃝ Not very useful or not useful at all ⃝ Difficult to say

**Lifestyle, keeping to a health-promoting lifestyle (exercise, diet and similar)**

⃝ Very useful ⃝ Somewhat useful ⃝ Not very useful or not useful at all ⃝ Difficult to say

**Reminders to take regular medications and vaccinations, and keep check-up appointments**

⃝ Very useful ⃝ Somewhat useful ⃝ Not very useful or not useful at all ⃝ Difficult to say

1. **Which of the following statements do you agree with regarding health apps and their use in treating patients with type 2 diabetes mellitus? (Tick as appropriate)**

⃝ Health apps do not provide adequate protection of sensitive data

⃝ Health apps raise motivation amongst patients to take a proactive approach towards their own health

⃝ Health apps empower patients by giving them more control over their health and health management

⃝ Health apps pose a substantial risk of misdiagnosis that may arise from inaccurate measurement, malfunction or similar

⃝ Health apps help in briefing patients on health and disease issues

⃝ Health apps are too complicated for many patients to use, especially older patients, which could result in false health data being collected with treatment failure in extreme cases

⃝ Health apps reinforce patient compliance

⃝ Additional information covered by health apps help physicians treat patients more effectively and personally

⃝ Health apps mean more rather than less of a workload for physicians due to the additional services required

**⃝** Health apps speed up the process of identifying diseases and disease risks

⃝ Health apps detract from the personal element in doctor-patient relationships

⃝ Health apps make consultation between physician and patient easier

1. **In what areas of application do your type 2 diabetes mellitus patients use health apps as far as you are aware? (Mark as appropriate)**

⃝ Prevention such as self-monitoring for risk factors (blood sugar, blood pressure, weight) and health data (steps taken, amounts drunk, diet, carbohydrate unit calculation, meal planning and similar)

⃝ Monitoring and management (documenting parameter trends, risk factors, and symptoms, such as by uploading photos and questionnaires, disease information, sequelae and disease management, medication tracking and calculation)

⃝ Lifestyle, keeping to a certain lifestyle (exercise, pedometry, diet and similar)

⃝ Reminders to take medication regularly, blood sugar measurement, checkups and prevention consultations

⃝ Don’t know, not informed

1. **How much of a role do health apps play in detecting or diagnosing disease more quickly in your opinion or experience? The role health apps play in diagnostics is:**

⃝ Very important ⃝ Rather important ⃝ Not so important ⃝ No benefit ⃝ Difficult to say or no health apps used

1. **Which of the following disease profiles could be more rapidly or effectively detected by health apps in your opinion or experience? (You may choose multiple options)**

⃝ Hypoglycaemia

⃝ Hyperosmolar coma

⃝ Diabetic foot syndrome

⃝ Metabolic syndrome

⃝ Diabetic retinopathy

⃝ Diabetic nephropathy

⃝ Diabetic neuropathy

⃝ Coronary heart disease

⃝ Depression

⃝ Other:

1. **Various positive effects on health are listed below. What results have you seen from your type 2 diabetes mellitus patients successfully using a health app? (You may choose multiple options)**

⃝ Increase in compliance (such as in taking medication, measuring blood sugar)

⃝ Weight reduction (such as BMI, abdominal circumference, waist circumference)

⃝ HbA1c decrease to below 7.5%

⃝ Regression of metabolic syndrome

⃝ Regression of psychological side effects (such as depression)

⃝ Prevention of sequelae (such as diabetic foot syndrome, CHD)

⃝ Decrease in complications (such as hypoglycaemia)

⃝ Elimination of need for more severe management options (such as insulin therapy)

⃝ Complete recovery

⃝ Other:

1. **How would you rate your awareness and knowhow regarding the general range of health apps available for type 2 diabetes mellitus prevention and/or management?**

⃝ Very extensive ⃝ Rather extensive ⃝ Rather restricted ⃝ Very restricted

1. **How would you rate your awareness and knowhow at distinguishing good from bad health apps for type 2 diabetes mellitus prevention and/or management?**

⃝ Very extensive ⃝ Rather extensive ⃝ Rather restricted ⃝ Very restricted

1. **How would you rate your awareness and knowhow at advising your patients on health apps for type 2 diabetes mellitus prevention and/or management?**

⃝ Very extensive ⃝ Rather extensive ⃝ Rather restricted ⃝ Very restricted

1. **Some suggestions for future improvement in type 2 diabetes mellitus health apps are listed below. Which three suggestions do you think are most important? (You may make up to three choices)**

⃝ Legal definition of authoritative quality criteria in health apps to ensure trustworthiness

⃝ Definition of authoritative data privacy standards for health apps to ensure consumer and patient protection

⃝ Obligation of providers to have their new health apps certified before they reach the market

⃝ Option to prescribe health apps shown to contribute to healthcare and/or disease management

⃝ Physicians should not have to risk liability, such as medical malpractice suits, arising from bugs in a health app

⃝ Insurance policy holders from all public health insurance organisations should receive bonuses or bonus programmes for using certain health apps regularly and transferring the data to the statutory health insurance organisation

⃝ The physicians’ fee schedule should include a position for medical services involving health apps (such as a special consultation number)

⃝ Inclusion of health apps for use in patient care according to the national type 2 diabetes mellitus care guideline

⃝ Other, namely:

1. **Assuming the national type 2 diabetes mellitus care guideline specifically addressed health apps and their use in disease prevention, monitoring and management, that is, assuming they provided detailed recommendations and instructions on this issue: Would you then be willing to use health apps in patient care more than before?**

⃝ Yes, much more ⃝ Yes, somewhat more ⃝ No

1. **Which guidelines are useful in type 2 diabetes mellitus management in your opinion or experience? (You may choose multiple options)**

⃝ National care guideline

⃝ German Diabetes Association (DDG)

⃝ German internal medicine association (DGIM)

⃝ German College of General Practitioners and Family Physicians (DEGAM)

⃝ International guidelines (such as European and US guidelines)

⃝ Periodicals and textbooks (such as Deutsches Ärzteblatt [medical journal])

⃝ Other, namely:

We’re almost done. Finally, we would like to ask you for some sociodemographic data. Like the rest of the questionnaire, the information you give here will of course be treated in strict confidence and anonymity.

**You are…**

⃝ Male ⃝ Female ⃝ Diverse

Your **age**:

**Where is your medical practice located?** In a municipality or city with a population of…

⃝ More than 100,000 ⃝ 20,000 to 100,000 ⃝ 5,000 to 20,000 ⃝ less than 5,000 inhabitants

**Which model** most accurately describes your medical practice?

⃝ Individual practice (you are the only physician)

⃝ Individual practice with employed physicians

⃝ Joint practice

⃝ Group practice

⃝ Other form of medical practice

**How many patients** come to your practice for treatment each quarter?

⃝ 500 to 750 ⃝ 751 to 1,000 ⃝ 1,001 to 1,500 ⃝ 1,501 to 2,000 ⃝ More than 2,000

**Thank you for your time!**

**Please use the enclosed SAE if you have filled out this survey in paper form.**

Is there anything else you would like to tell us?

Please use the space for suggestions, comments, and criticism.
