# Supplementary material for: Attitudes and experiences of registered diabetes specialists in using health apps for managing type 2 diabetes: results from a mixed-methods study in Germany 2021/2022
Source: Arch Public Health. 2023 Mar 7;81:36. doi: 10.1186/s13690-023-01051-0 (PMC9990333; doi:10.1186/s13690-023-01051-0)
Supplement: Supplementary file 2 — Additional file 2. Interview guideline. [file 13690_2023_1051_MOESM2_ESM.docx]

**Additional file 2: Interview guideline**

*Clinical picture of type 2 diabetes and its significance in everyday practice*

If you look at your patient base: how important is type 2 diabetes mellitus as a clinical picture?

To what extent has the number of patients affected by this clinical picture increased? Where do you see the reasons for this?

*Perception and significance of health apps in general*

What do you think of health apps in general?

Where do you see opportunities and strengths, where challenges and risks of health apps? Do the benefits outweigh the problems?

To what extent have you already had experience with health apps in the context of your own patient care? Where and in which areas?

*Health apps with regard to type 2 diabetes mellitus*

There is a wide range of health apps especially for type 2 diabetes mellitus. To what extent is the use of health apps well suited for this clinical picture? Do you have the impression that apps offer added value in the prevention, follow-up and/or therapy of type 2 diabetes mellitus?

In which areas of application do you think health apps are particularly useful and can they have a positive effect, especially on patients with type 2 diabetes mellitus?

Which clinical pictures/consequential damage could be uncovered faster and more effectively through the use of health apps?

*Own experiences using type 2 diabetes health apps*

What is your experience in the area of prevention, monitoring and therapy for patients with type 2 diabetes mellitus? Where have you observed positive and where rather negative effects? What positive effects have you observed as a result of successful app use in your patients with type 2 diabetes mellitus?

In which application areas of your patients with type 2 diabetes mellitus are health apps used?

Do you ever talk to patients with type 2 diabetes mellitus about health apps, or are you more likely to be asked about them? Do you ever make specific recommendations?

What criteria are important to you when it comes to recommending a specific health app to patients? What requirements does such an application have to meet?

Do you occasionally find out about health apps specifically for the clinical picture of type 2 diabetes mellitus? If so, what sources do you use? What is the significance of information and overview sites on the Internet? Which sites do you use?

In your opinion, what should be improved about health apps in the future in order to see a greater use of health apps with regard to diagnostics, therapeutic success and prevention in patients with type 2 diabetes mellitus?

How do you rate your knowledge and competence when it comes to overviewing the range of available health apps for the prevention and/or treatment of type 2 diabetes mellitus or distinguishing between good and bad health apps or patients to advise on this? How do you rate this for diabetology practices in general?

Which clinical guideline(s) do you follow with regard to the treatment of patients with type 2 diabetes mellitus?

Assuming that new clinical guidelines on the clinical picture of type 2 diabetes mellitus specifically address the use of health apps and their use for prevention, disease monitoring and therapy and provide detailed recommendations and instructions on this. Given this, would you be willing to use health apps more than in the past in patient care?
